# The Transactional Relationship between Parental and Adolescent Depressive Symptoms: The Mediating Effect of Nurturant–Involved Parenting

**DOI:** 10.3390/ijerph17218240

**Published:** 2020-11-07

**Authors:** Jinqin Hou, Zhiyan Chen, Fei Guo

**Affiliations:** 1 National Institute of Education Sciences, Beijing 100088, China; houjq@nies.net.cn; 2 CAS Key Laboratory of Mental Health, Institute of Psychology, Beijing 100101, China; chenzy@psych.ac.cn

**Keywords:** depressive symptoms, transactional relationship, child-driven effect, parenting, adolescents

## Abstract

Sameroff’s transactional theory emphasizes a bidirectional process between parents and offspring. The present study explored the reciprocal relationships between parental and adolescent depressive symptoms using a cross-lagged model and examined the mediating effect of nurturant–involved parenting on the relationship between them. Data for the present study were collected from a longitudinal study, and a total of 1644 adolescents and their mothers and fathers participated in the present study. The results revealed a reciprocal relationship between maternal and adolescent depressive symptoms, and the child-driven effect was more robust than the mother-driven effect. Adolescent depressive symptoms significantly predicted paternal depressive symptoms, but not vice versa. In addition, adolescent depressive symptoms indirectly predicted maternal and paternal depressive symptoms by deteriorating nurturant-involved parenting. These findings highlight a child-driven effect on parents’ psychopathology, which may shed light on the mechanism underlying depression transmission between parents and adolescents.

## 1. Introduction

There is burgeoning evidence that depressive symptoms increase sharply during adolescence [1,2]. The high prevalence rates of depression during adolescence makes this developmental stage of great interest for depressive symptoms. Since depression can be transmitted intergenerationally [3], the effect of parental depressive symptoms on adolescents’ depressive symptoms has drawn much attention. Mounting evidence from genetic studies has indicated that depressive symptoms are heritable [4]. However, besides genetic influence, it has been suggested that environmental factors also play a vital role in the intergenerational transmission of parental depressive symptoms to their offspring’s depressive symptoms [5,6]. Since both genetic and environmental influences account for the association between parental and adolescent depressive symptoms, it is theoretically possible that adolescents’ depressive symptoms may influence parental depressive symptoms due to the evocative gene–environment correlations (evocative rGE), which happens when adolescents’ genetically-influenced traits or symptoms (e.g., depression) elicit environmental responses from others [7,8], which may in turn be associated with elevated parental depressive symptoms. However, few studies have explored the effect of child depressive symptoms on parental depressive symptoms; i.e., the “child-driven” effect. It has recently been suggested that, when studying the intergenerational transmission of risk for depression, researchers need to recognize that children are not simply passive recipients of their environment [4]. The present study used a transactional model to explore the reciprocal relationship between parental and adolescent depressive symptoms, conducting a cross-lagged analysis and examining the mediating effect of parenting practices (nurturant–involved parenting) on the relationship between them.

Accumulating evidence has suggested that parental depression is detrimental for adolescents’ development. Maternal depression has been shown to be significantly related to higher levels of internalizing and externalizing problems and a more negative affect/behavior [9]. As the designated primary caregiver, most research in this area has focused on mothers [10]. Research has indicated that maternal depressive symptoms are not only the source of a set of risk factors but also predict the trajectory of the growth of adolescents’ depressive symptoms [11]. When maternal depressive symptoms become more or less severe, children’s behavioral problems increase or decrease reciprocally [12].

In the past decade, researchers have realized that fathers also play an important role in their offspring’s development [13,14]. However, more research is needed to achieve the consistency in findings necessary to concretely establish the effect of paternal psychopathology on children. Some studies have overlooked the association between paternal internalizing symptoms and offspring internalizing symptoms [15], while others have indicated that paternal psychopathology predicts offspring depression, with no difference between fathers and mothers. However, such an effect could be weaker if rated by cross-raters rather than by with-raters [12]. At the same time, another study found that fathers’ depressive symptoms were related with boys‘ but not girls’ depressive symptoms [16]. These inconsistent findings remind researchers that the relationship between paternal depression and offspring depression is worthy of further study, especially considering factors such as raters and the gender of offspring.

In traditional Chinese culture, with the father–child relationship representing the archetype of social order in Confucian ethics [17], fathers are expected to distance themselves from daily child care and nurturance. Their responsibilities mainly focused on the aim of being a strict yet benevolent disciplinarian and moral role model [18]. During everyday interactions with their children, fathers are discouraged from providing explicit verbal expressions of warmth and concern [19]. However, Chinese society has been undergoing profound socio-cultural changes (e.g., dramatic economic growth and the one-child policy). Although in contemporary China, fathers are still less emotionally warm than mothers [20] and take less responsibility for the emotional care of children than mothers [21], the rigidity of the traditional “strict father, kind mother” pattern has reduced [22]. Some studies have shown that, among couples with inter-parental parenting differences, a common pattern was found that was named “tiger moms, panda dads”, which indicated that fathers were less controlling and more supportive than mothers [23]. The present study therefore examined not only mother–adolescent dyads but also father–adolescent dyad models.

Contemporary theories of developmental psychopathology emphasize reciprocal and transactional processes of change; e.g., Sameroff’s transaction model [24,25], which focuses on the complex influence that child behaviors impose on parental behaviors and vice versa. Transactional processes are coincident with Sameroff’s model in terms of underlining the child-driven effect, which has been examined extensively in the context of parenting behaviors and child-externalizing behavioral problems [26,27]. Although less is known about the effect of child behavioral problems on parental psychopathology, evidence suggests that children’s behavioral problems are likely linked with parental depression [28,29]. However, much less is known about the bidirectional relationship between child depressive symptoms and parental depressive symptoms. To our knowledge, only a few studies have investigated transactional associations between parental psychopathology and child-internalizing problems. These studies provide a mixed picture on the subject. For example, Brooker et al. [30] and Hails et al. [31] identified transactional associations between parental internalizing symptoms and child-internalizing problems in infants and early childhood; however, other studies did not show bidirectional associations between childhood and parental psychopathology in preschoolers [32].

Since the parent–child interaction is reciprocal, one question remains: are parent-to-child associations stronger than child-to-parent associations or vice versa? Larsson and colleagues [33] have found that the association between parenting and childhood antisocial behavior could be best explained by both parent-driven and child-driven effects in toddlers at the ages of 4 and 7 years. However, Hipwell et al. [34], who examined the reciprocal relationship between parenting and childhood behavior in girls aged 7 to 12 years, suggested that the direction of prediction from girls’ behaviors to parental depression was stronger than in the other direction. A possible reason for this inconsistency in previous studies is the ages of the participants. It is well known that adolescence is a developmental period characterized by multiple changes in virtually every aspect of an individual’s life. Physiological and cognitive developments foster an increasingly egalitarian view of relationships that were previously oriented around the unilateral authority of parents, and adolescents increasingly aspire to reciprocity and equal power in their interactions with their parents [35]. As a result, the child-driven effect may become more evident when entering adolescence.

Theory and research suggest that the environment—especially familial factors—plays an important role in influencing individual development. For example, in bioecological systems theory, as a microsystem context, the family is where development occurs. Nurturant–involved parenting is defined as being characterized by high warmth, emotional support, positive communication, low hostility and low rejection [36], which indicates low conflict and close parent–child relationships [37,38]. The existing literature has identified nurturant–involved parenting as one of the most robust protectors against externalizing and internalizing problems in children and adolescents [39,40,41], and those results have been consistent across cultures [42,43], including the Chinese context [44]. Specifically, Goodman and Gotlib [45] have suggested that it is difficult for depressed parents to follow a warm and supportive parenting style; conversely, depression is highly associated with inconsistent, insensitive and inattentive parenting, which contributes significantly to the development of psychopathology in children. As pertains to issue of the parent-driven effect on the adjustment of the relationship between parents and offspring, Johnson et al. [46] found that maladaptive parenting mediates the relationship between parental psychological disorders and those of their offspring in late adolescence and early adulthood. Elgar et al.’s study [47] showed evidence of mediating effects of both positive and negative domains of parental behaviors; reductions in parental nurturance and monitoring and increases in parental rejection each carried a significant amount of influence on a child’s functioning, which were a possible result of parents’ depressive symptoms.

Regarding the child-effect on the links between parental and adolescents’ adjustment, Raposa et al. [48] showed that child-related acute and chronic stress were mediators of the relationship between youth diagnoses and the presence of maternal depression at follow-up. Very few studies have examined the mediating effect of parenting in the relationship between parental and adolescent depressive symptoms from a child-driven perspective. To expand on previous research, one of the aims of present study was to explore the potential mediating role of nurturant–involved parenting. Examining the mechanisms by which the risk of depressive symptoms is transferred between adolescents and parents is important for understanding the nature of these problems. Previous studies have shown that parenting behaviors change with children’s symptoms. For example, Kim and colleagues [49] found that children whose symptoms increased over time reported increases in hostility and harsh–inconsistent parenting and decreases in warmth and nurturant–involved parenting. At the same time, research has suggested that the sense of efficacy of parenting is related to parental emotion problems [50]. According to the self-efficacy theory, which is concerned with the relationship between cognitions of self-devaluation, an inability to cope and depression [51], an increase in negative parenting or decrease in positive parenting is related to parents’ low sense of efficacy, and a low sense of efficacy is related to depression. Based on previous studies, the present study aimed to examine the mediating effect of parenting on the relationship between parental and adolescent depressive symptoms from a child-driven effect perspective.

### The Current Study

A recent meta-analysis based on 82,592 Chinese children showed that the pooled point prevalence of major depressive disorders (MDD) in children and adolescents was 1.3% in China [52] and comparable with the global prevalence of MDD (2.6%, CI 95% 1.7–3.9) in children and adolescents aged 6–18 years [53]. However, few studies have examined the relationship between parental depressive symptoms and adolescents’ depressive symptoms in the Chinese context.

The present study sought to examine the transactional interplay between maternal/paternal and adolescent depressive symptoms using a cross-lagged design and to explore the mechanisms by which the risk of depressive symptoms is transferred between parents and adolescents. First, the reciprocal relationships between maternal/paternal and adolescent depressive symptoms were examined separately. Mounting evidence has indicated that parental depressive symptoms—especially maternal—put adolescents at risk for impaired development. Extending this further, we expected the child-driven effect to be evident in the relationship between parental and adolescent depressive symptoms. Second, the mediating effect of nurturant–involved parenting was examined in the cross-lagged relationship between parental and adolescent depressive symptoms. We expected a mediating effect of parenting on the influence of adolescents’ depressive symptoms on parental depressive symptoms and hypothesized that adolescent depressive symptoms predict a decrease in maternal/paternal nurturant–involved parenting, which in turn predict maternal/paternal depressive symptoms. The present study has an important methodological strength, which is its use of multiple-informant data to test the hypotheses. It could be argued that findings regarding the intergenerational transmission of psychopathology may be inflated because of shared-rater variance [12], which typically occurs when all constructs are completed by a single rater. In the current study, each family member—the adolescent, the mother and the father—rated their own depressive symptoms. Regarding parenting, both parental and adolescent reports were included in the models by using a latent variable approach.

## 2. Materials and Methods

### 2.1. Participants and Procedures

The current sample was drawn from a longitudinal school-based study; i.e., the Chinese Adolescent Mental Health Research Program [54]. The data of the first wave (W1) were collected in 2008 and 2009. Schools were sampled from nine cities, which thus provided a broad coverage of four geographical regions and different city sizes in China, including one metropolis, four provincial capitals, and four mid-sized cities. Two schools in each city were randomly selected: one primary school and one middle school. For each selected school, one classroom was randomly sampled; the survey took place in these classrooms. Permission to conduct the research was obtained from the school administrators.

A year after obtaining the data, a longitudinal study was conducted. Because of the difficulty in contacting students who graduated, only those who were not in the graduating class (i.e., grades 4–5, 7–8 and 10–11 at W1) were included in the current school-based longitudinal study. The attrition from W1 to wave 2 (W2) was 24.8%, and there were no differences in depressive symptoms between participants and non-participants in W2 in grades 4, 7 and 10. However, those who participated showed lower levels of depressive symptoms than those who dropped out at W2 in grade 5 (*t* = 3.10, *p* < 0.05), grade 8 (*t* = 3.10, *p* < 0.05) and grade 11 (*t* = 2.29, *p* < 0.05; all in the graduating class at W2).

At wave 3 (W3), those students who were not in the graduating class at W2 (i.e., grades 4, 7 and 10 at W1) were included in the third-year follow-up school-based study. The attrition from W2 to W3 was 38.6%, and no substantial differences emerged in depressive symptoms between those who participated and those who dropped out at W3 (*t* = 1.23, *p* > 0.05)

In addition to the school-based study, to enlarge the longitudinal sample, a home-based study that aimed to invite students who graduated from schools at W2 and W3 was conducted. As a result, 427 graduates (i.e., grades 6, 8 and 12 at W1) participated in W2, and there were no differences in depressive symptoms between graduates who participated and those who did not participate in W2 (*t* = 1.74, *p* > 0.05). At W3, 563 graduates at W2 (grades 5, 7 and 11 at W1) completed the assessments in W3, and there were no differences in depressive symptoms between graduate students who participated and those who did not participate in W3 (*t* = 1.12, *p* > 0.05).

The final longitudinal sample consisted of 785 boys and 859 girls born between 1992 and 1998; the average ages at W1 were 12.99 ± 1.84 for boys and 12.96 ± 1.87 for girls. Meanwhile, the average age for mothers was 39.09 ± 4.54 years and that for fathers was 40.63 ± 4.69 years at W1. Approximately 60% of parents had an education level higher than senior high school.

### 2.2. Measures

#### 2.2.1. Depressive Symptoms

The Center for Epidemiological Studies Depression Scale (CES-D) [55] was used to assess depressive symptoms. The CES-D has been widely used with Chinese samples [56], and a short version (CES-D-13) has been validated and used for Chinese adolescents [34]. The participants reported the frequency at which their depressive symptoms had occurred during the past week on a four-point Likert scale ranging from “rarely or none of the time” to “most of or all of the time.” In the present study, at each wave, mothers, fathers and adolescents reported their own depressive symptoms. The Cronbach’s alphas for the youth were 0.84–0.88 in three waves, and for mothers and fathers, these values were 0.84–0.85 and 0.83–0.85 at waves 2 and 3, respectively.

#### 2.2.2. Nurturant-Involved Parenting

Nurturant–involved parenting was measured using scales adapted from the Iowa Youth and Families Project [57,58]. Using a scale ranging from 1 (never) to 5 (always), a summed composite of 12 items indexed the degree to which parents knew about their children’s whereabouts and activities, as well as the extent to which mothers and fathers used reasoning and open communication with adolescents. The Cronbach’s alphas for the youth reports (α = 0.80–0.90 for maternal parenting; α = 0.85–0.88 for paternal parenting), mothers (α = 0.87–0.92) and fathers (α = 0.86–0.92) were adequate for both waves 2 and 3 in the present study.

### 2.3. Data Analysis

Cross-lagged path analysis is widely used to infer directional relationships between variables in studies with longitudinal designs. This type of model allows for a simultaneous examination of the longitudinal influences of one variable on another, and vice versa, while controlling for the stability of each variable over time. The cross-wave, within-trait coefficients indicate the stability of depressive symptoms over time while controlling for cross-lagged contributions from each other. The cross-lagged coefficients allowed us to determine whether the association between parental and adolescent depressive symptoms was parent-driven or child-driven. To examine the potential mediating role of nurturant–involved parenting, a cross-lagged mediating model was tested. In the present study, nurturant–involved parenting was reported by both parents (mothers and fathers) and adolescents. Because structural equation modeling (SEM) techniques allow the examination of latent variables, these techniques are ideally suited for testing multi-informant variables. In this study, we tested the transactional model and mediational hypotheses with longitudinal data, using the SEM software AMOS 23.0 (IBM Corp., Armonk, NY, USA) [59]. Data cleaning and descriptive statistics were conducted using SPSS software (version 24, IBM Corp., Armonk, NY, USA). Preliminary data cleaning analyses were done to identify and correct any errors in data collection or recording. The distribution of variables was assessed for normality through skew and kurtosis, and all variables approximated a normal distribution. Meanwhile, the full information maximum likelihood (FIML) raw data technique was used to account for missing data [60].

## 3. Results

### 3.1. Preliminary Analyses

Bivariate relationships were relatively stable within each domain over time (see Table 1 and Table 2). The adolescents’ depressive symptoms at each year were positively and significantly correlated with one another both among girls and boys. The parents’ depressive symptoms (both mothers’ and fathers’) were also stable over time. Both the adolescents’ and parents’ depressive symptoms were significantly correlated at each time point and across waves. The correlations between boys’ and maternal depressive symptoms were 0.12 to 0.25 (*p* < 0.01), and those between the boys’ and paternal depressive symptoms were 0.13 to 0.21 (*p* < 0.01); meanwhile, the correlations between girls’ and maternal depressive symptoms were 0.12 to 0.22 (*p* < 0.01), while the correlations were 0.09 (*p* < 0.05) to 0.18 (*p* < 0.01) with fathers’ symptoms. Bivariate correlations across parents’ reports and adolescent reports on parenting were robust (*r =* 0.40–0.63, *p* < 0.01). In general, adolescents’ depressive symptoms were also negatively associated with parental nurturant–involved parenting.

### 3.2. The Cross-Lagged Model

**Figure 1:**
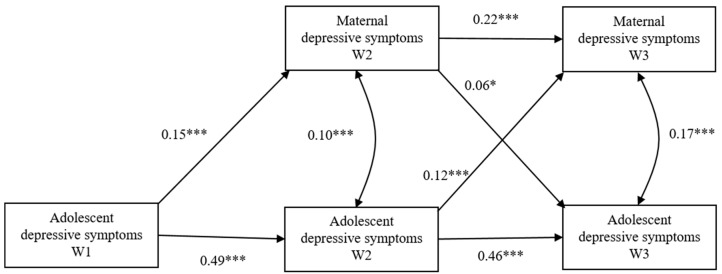
The cross-lagged model for maternal depressive symptoms and adolescent depressive symptoms. * *p* < 0.05, *** *p* < 0.001.

A structural equation model was used to assess cross-lagged associations between parent and adolescent depressive symptoms after controlling for initial adolescent symptom levels (see Figure 1). The model was evaluated separately for mother–adolescent and father–adolescent dyads. For mother–adolescent dyads, the model was reasonably adequate (*χ*^2^ = 25.98, *df* = 2, comparative fit index (CFI) = 0.98, root mean square error of approximation (RMSEA) = 0.085). The coefficients presented in Figure 1 for the cross-lagged paths indicate both child-driven and parent-driven effects. From W1 to W3, reciprocal relationships between mothers’ and adolescents’ depressive symptoms were observed: more adolescent depressive symptoms at W1 were associated with greater maternal depressive symptoms at W2 (*β* = 0.15, *p* < 0.001), and more adolescent depressive symptoms at W2 were associated with an increase in maternal symptoms at W3 (*β* = 0.12, *p* < 0.001); at the same time, greater maternal depressive symptoms at W2 led to more adolescent depressive symptoms at W3 (*β* = 0.06, *p* < 0.05). In addition, results showed that child-driven effects were more robust than parent-driven effects. The gender effect was not evident (Δ*χ*^2^ = 4.24, *p* > 0.05).

**Figure 2:**
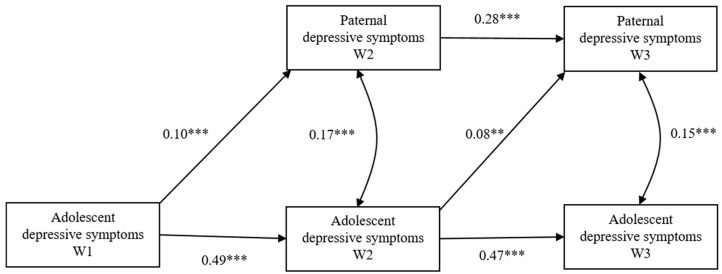
The cross-lagged model between paternal depressive symptoms and adolescent depressive symptoms. ** *p* < 0.01, *** *p* < 0.001.

For the father–adolescent model (see Figure 2), the fit indices indicated an acceptable fit (*χ*^2^ = 24.15, *df* = 3, CFI = 0.98, RMSEA = 0.07). In this model, only the child-driven effect was observed; adolescent depressive symptoms at W1 significantly predicted paternal depressive symptoms at W2 (*β* = 0.10, *p* < 0.001), and greater adolescent symptoms at W2 led to more paternal depressive symptoms at W3 (*β* = 0.08, *p* < 0.005). No gender difference was found (Δ*χ*^2^ = 11.34, *p* > 0.05).

### 3.3. Mediation Model for Parenting

**Figure 3:**
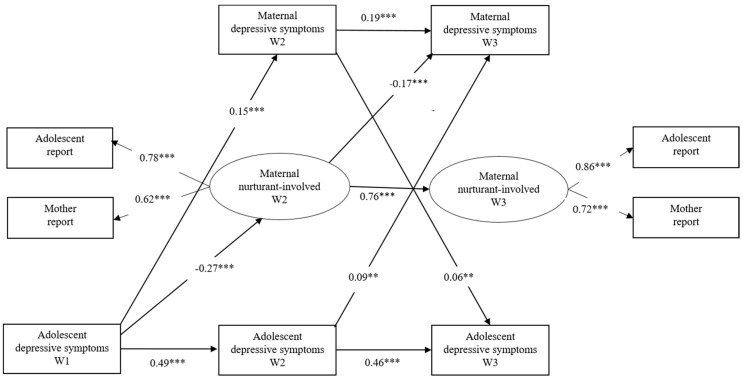
Transactional effects between adolescents’ depressive symptoms, maternal nurturant–involved parenting and maternal depressive symptoms. Maternal nurturant–involved = maternal nurturant–involved parenting. ** *p* < 0.01, *** *p* < 0.001.

To evaluate the mediating role of parental nurturant–involved parenting, two latent variables—nurturant–involved parenting at W2 and nurturant–involved parenting at W3 (adolescent report and mother/father report)—were added to the previous models, and the following associations were tested: between adolescent depressive symptoms at W1 and nurturant–involved parenting at W2, between adolescent depressive symptoms at W2 and nurturant–involved parenting at W3, between nurturant–involved parenting at W2 and parental depressive symptoms at W3, between nurturant–involved parenting at W2 and adolescent depressive symptoms at W3, and between parental depressive symptoms at W2 and nurturant–involved parenting at W3 (see Figure 3). This mediation model was also evaluated separately for mother–adolescent and father–adolescent dyads.

For mother–adolescent dyads, the model demonstrated an acceptable fit (*χ^2^* = 196.06, *df* = 17, CFI = 0.94, RMSEA = 0.08). As Figure 3 shows, on the one hand, adolescents’ depressive symptoms predicted maternal depressive symptoms directly (*β* = 0.15, *p* < 0.001 from W1 to W2; *β* = 0.09, *p* < 0.01 from W2 to W3). On the other hand, adolescents’ depressive symptoms at W1 predicted maternal depressive symptoms at W3 indirectly through the mother’s nurturant–involved parenting. Higher levels of adolescent depressive symptoms at W1 predicted lower levels of maternal nurturant–involved parenting at W2 (*β* = −0.27, *p* < 0.001), which predicted greater maternal depressive symptoms at W3 (*β* = −0.17, *p* < 0.001). At the same time, the parent-driven effect was still significant; that is, maternal depressive symptoms at W2 predicted adolescents’ depressive symptoms at W3 (*β* = 0.06, *p* < 0.01).

**Figure 4:**
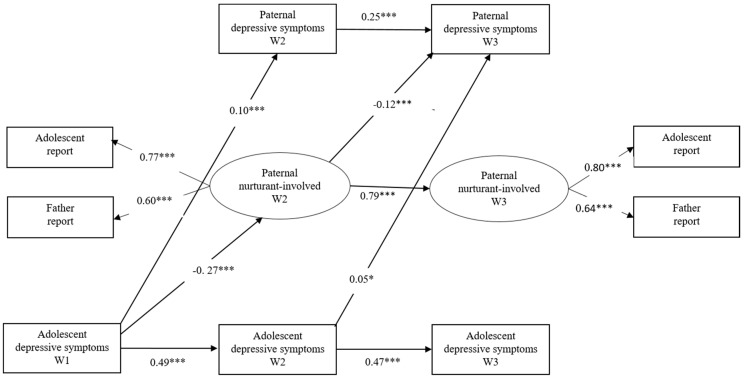
Transactional effects between adolescents’ depressive symptoms, paternal nurturant–involved parenting and paternal depressive symptoms. Paternal nurturant–involved = paternal nurturant–involved parenting. * *p* < 0.05, *** *p* < 0.001.

For the father–adolescent model (see Figure 4), the fit indices showed an acceptable fit (*χ^2^* = 219.95, *df =* 18, CFI = 0.92, RMSEA = 0.08). The pattern of the transactional relationships between variables shared some similarities with the mother–adolescent model. On the one hand, adolescents’ depressive symptoms predicted paternal depressive symptoms directly (*β* = 0.10, *p* < 0.001 from W1 to W2; *β* = 0.05, *p* < 0.05 from W2 to W3); on the other hand, adolescent depressive symptoms influenced paternal depressive symptoms by deteriorating fathers’ nurturant–involved parenting. Specifically, adolescent depressive symptoms at W1 negatively predicted paternal nurturant–involved parenting at W2 (*β* = −0.27, *p* < 0.001), and paternal nurturant–involved parenting negatively predicted paternal depressive symptoms at W3 (*β* = −0.12, *p* < 0.001).

## 4. Discussion

Contemporary theories of developmental psychopathology and family processes emphasize the reciprocal and transactional processes of change among family members [61,62]. Methodological development has allowed researchers to explore the underlying mechanisms of these theories in a sophisticated manner [63]. This study contributes to the understanding of the association between parental and adolescent depressive symptoms by considering child-driven effects as well as by exploring the mechanism of child-driven effects on parental depressive symptoms by examining the mediating roles of nurturant–involved parenting. To our knowledge, this study is one of the first to attempt to apply a longitudinal design to examine the transactional interplay between parental and adolescent depressive symptoms in China. The cross-lagged analyses revealed evidence in support of the presence of child-driven effects. Moreover, this effect was shown to be mediated by nurturant–involved parenting. Additionally, although depressive symptoms have their own endogenous sources of stability, the present study suggested that stability may also be maintained in part via a cumulative continuity process [64] in which early depressive symptoms deteriorate future parenting, which in turn increases the likelihood of developing future depressive symptoms, especially in connection with interactions with their mothers.

Consistent with our hypothesis, besides the intergenerational transmission from mothers to adolescents, adolescent depressive symptoms were also shown to influence parental depressive symptoms. Maternal depressive symptoms significantly predicted adolescents’ depressive symptoms, while those of fathers did not—a finding consistent with several previous studies [15,65,66] but in contrast with others [67] that did not find that the amount to which paternal psychopathology predicts child-internalizing problems differed between mothers and fathers. Some study only found the child-driven effect within raters of parent and offspring psychopathology but not across raters. The child-driven effect might reflect shared-rater variance [12]. However, in the present study, significant child-driven effects were detected when multiple-informant data were used, which showed that the effects were robust and could not be explained by shared-rater variance. The inconsistent findings may relate to the different ages of children in the two studies. As some study have shown, the child-driven effect becomes more obvious as children grow up [8].

The result that paternal depressive symptoms were not found to predict adolescent depressive symptoms may be influenced by the different roles played by mothers and fathers in their adolescents’ development. As the first caregiver in one’s life, mothers are generally more involved in the development of their offspring’s emotions and feelings, whereas fathers are generally more involved in the development of their autonomy and independence [68]. In traditional Chinese culture, the father serves as the breadwinner of the family and is more responsible for dealing with external issues outside of the family [69]. Compared to mothers, fathers may be more emotionally detached and distant from children [70]. Although several recent studies have suggested ongoing changes in Chinese fathering styles and reductions in the differentiation of parental roles as prescribed by social norms [23], Chinese fathers are still generally less involved in daily childrearing, especially regarding old children [71]. The effects of fathers’ roles as parents are acknowledged; however, some prior studies have shown that fathers may have less of an influence on children’s developmental outcomes than mothers. For example, in a study based on a nationally representative dataset of Chinese adolescents, the absence of a father was found to have little or no effect on adolescents’ test scores and depressive symptoms, whereas living in mother-absent households was significantly associated with those negative outcomes [72]. These between-parent differences findings in the present study are also consistent with previous research works in other cultures [37].

Pertaining to the relative impact of parent-driven and child-driven effects, the child-driven effect was more obvious in this study. Previous findings have suggested that the magnitude of these effects varied as a function of the age of the child [8,63]. As children grow older, physical maturation, cognitive ability development and changes in the children’s social environment make youths assert more autonomy in their interactions with their parents; therefore, the child-driven effect increases in magnitude during the transition from childhood to adolescence [73,74]. In the present study, child-driven effects were also evident in the interplay between maternal and adolescent depressive symptoms.

Expanding on past research, this study found indirect effects of adolescent depressive symptoms on parental depressive symptoms. Consistent with the evolutionary perspectives which assume that the basis of a depressed mood is interpersonal [75], the present study suggests that, with increases in depressed mood, social dysfunction may affect and deteriorate parent–child interaction, which in turn can elicit parental depressive symptoms. Specifically, adolescent depressive symptoms cause a decrease in parents’ nurturant–involved parenting, which in turn worsens parental depressive symptoms. Moreover, under the influence of the parent-driven effect, interactions between adolescents and mothers are made even more difficult since maternal depressive symptoms could further strengthen adolescents’ depressive symptoms. Therefore, adolescent depressive symptoms, maternal depressive symptoms and low nurturant–involved parenting affect each other in a vicious circle. These findings are consistent with the widely acknowledged view that children’s characteristics can influence children’s development by eliciting specific parental behavior, which in turn influences subsequent child behaviors and emotions [76,77].

In addition to the four mechanisms of the parent-driven effect suggested by Goodman and Gotlib [45] in terms of genes and the environment, a gene–environment correlation may be another mechanism underlying child-driven effects; that is, the occurrence of a child-driven effect in the parent–child interaction may be caused by the evocative gene–environment correlation, which occurs when an individual’s behavior evokes an environmental response [78,79]. In other words, the genetic propensity to depression that adolescents inherit from their parents may put them at a high risk of being exposed to low nurturant–involved parenting. In the child-driven effect, there is not only a genetic pathway but also an environmental pathway. Findings from studies on adoption, children of twins and in vitro fertilization study have shown evidence that the environment plays an important role in the associations between parental and offspring depressive symptoms [5,80,81]. A meta-analysis showed that around 23% of the variance in individual differences in parental behavior in their sample could be explained in terms of evocative gene–environment correlations, indicating that the genetically influenced behaviors of a child evoke certain behaviors in parents and attest to the role that the child plays in shaping parenting; shared and non-shared environmental influences accounted for 43% and 34% of the variance in individual differences in parenting, respectively [82].

The following caveats should be taken into consideration when generalizing the current findings to other samples. First, students in graduating classes who participated in W2 showed a lower level of depressive symptoms than those who did not. This may be because some students in graduating classes dropped out of school, and adolescents who drop out of school have been shown to have higher levels of depressive symptoms than those who remained at school at the same age [83]. Whether the findings in the present study can be generalized to adolescents who have already dropped out of school needs to be studied further. Second, only one specific parenting practice was examined as the mediator in the present study. To expand the knowledge of the mechanism underlining the reciprocal relationships between parent and adolescent internalizing symptoms, future research may further elucidate other mediating and moderating factors, including the role of both parenting and adolescent traits.

Notwithstanding these limitations, the current study has a number of strengths and makes a contribution to the current literature. It is one of the few existing studies to have examined the bidirectional relationships in parental and adolescent depressive symptoms and explored parenting practice as a mediator of these relationships. It the present study, both mothers and fathers were included, and the finding of inter-parent differences is also noteworthy. This confirmed the necessity to examine the uniqueness of mothers and fathers. In addition, the multi-informant approach provided more robust evidence of the child-driven effect in the transmission of internalizing symptoms in the family than research utilizing single informants. Finally, the findings showed the role that nurturant–involved parenting plays in the reciprocal relationships between parental and adolescent depressive symptoms, identifying the potential processes that deteriorate or alleviate problems. It may be helpful to promote this parenting practice in the prevention and intervention of depression.

## 5. Conclusions

The findings in the current study are consistent with established conceptualizations regarding the origins of the coercive vicious cycle in which parental depressive symptoms, negative parenting practices and adolescent depressive symptoms affect each other. In particular, evidence from the present study demonstrates a child-driven effect on that downward spiral, advancing a framework for future research in this area. The child-driven effect deserves greater attention in both research and practice. Our findings highlight the importance of training for parents to reduce negative parenting, especially in families with a high risk of depression.

## Figures and Tables

**Table 1:** Descriptive statistics for the study variables (mother–adolescent) by adolescents’ gender.

Variable		1	2	3	4	5	6	7	8	9
1	W1 Adolescent depressive symptoms	—	0.47 **	0.31 **	0.12 **	0.14 **	−0.14 **	−0.26 **	−0.14 **	−0.23 **
2	W2 Adolescent depressive symptoms	0.52 **	—	0.48 **	0.16 **	0.14 **	−0.07	−0.25 **	−0.11 **	−0.16 **
3	W3 Adolescent depressive symptoms	0.33 **	0.47 **	—	0.14 **	0.22 **	−0.03	−0.15 **	−0.15 **	−0.23 **
4	W2 Maternal depressive symptoms	0.17 **	0.15 **	0.12 **	—	0.23 **	−0.33 **	−0.12 **	−0.16 **	−0.08 *
5	W3 Maternal depressive symptoms	0.24 **	0.20 **	0.25 **	0.24 **	—	−0.12 **	−0.16 **	−0.29 **	−0.22 **
6	W2 Ma. nurturant–involved (M)	−0.11 **	−0.04	−0.07	−0.24 **	−0.10 *	—	0.51 **	0.41 **	0.36 **
7	W2 Ma. nurturant–involved (A)	−0.23 **	−0.20 **	−0.12 **	−0.11 **	−0.23 **	0.43 **	—	0.43 **	0.60 **
8	W3 Ma. nurturant–involved (M)	−0.15 **	−0.10 *	−0.10 *	−0.15 **	−0.28 **	0.45 **	0.33 **	—	0.63 **
9	W3 Ma. nurturant–involved (A)	−0.14 **	−0.16 **	−0.26 **	−0.13 **	−0.24 **	0.36 **	0.45 **	0.56 **	—
Boys *M (SD)*		7.97 (6.02)	9.00 (7.08)	10.18 (7.06)	7.06 (5.85)	7.91 (6.02)	3.91 (0.79)	3.61 (0.85)	3.74 (0.81)	3.51 (0.83)
Girls *M* (*SD)*		8.11 (6.23)	9.75 (7.48)	10.16 (7.58)	6.80 (5.81)	7.53 (6.50)	3.97 (0.73)	3.81 (0.82)	3.98 (0.71)	3.79 (0.80)

Note. Ma. nurturant–involved = maternal nurturant–involved parenting; (M) = mother report; (A) = adolescent report; Lower diagonal = correlation matrix for the data of boys; Upper diagonal = correlation matrix for the data of girls; * *p* < 0.05, ** *p* < 0.01.

**Table 2:** Descriptive statistics for the study variables (father–adolescent) by adolescents’ gender.

Variable		1	2	3	4	5	6	7	8	9
1	W1 Adolescent depressive symptoms	—	0.47 **	0.31 **	0.03	0.11 **	−0.17 **	−0.24 **	−0.17 **	−0.18 **
2	W2 Adolescent depressive symptoms	0.52 **	—	0.48 **	0.18 **	0.15 **	−0.13 **	−0.28 **	−0.14 **	−0.20 **
3	W3 Adolescent depressive symptoms	0.33 **	0.47 **	—	0.09 *	0.17 **	−0.06	−0.20 **	−0.17 **	−0.31 **
4	W2 Paternal depressive symptoms	0.15 **	0.20 **	0.14 **	—	0.32 **	−0.26 **	−0.12 **	−0.17 **	−0.06
5	W3 Paternal depressive symptoms	0.17 **	0.13 **	0.21 **	0.22 **	—	−0.14 **	−0.17 **	−0.33 **	−0.19 **
6	W2 Pa. nurturant–involved (F)	−0.10 *	−0.08 *	−0.07	−0.29 **	−0.10 *	—	0.49 **	0.44 **	0.39 **
7	W2 Pa. nurturant–involved (A)	−0.23 **	−0.24 **	−0.15 **	−0.14 **	−0.13 **	0.40 **	—	0.38 **	0.57 **
8	W3 Pa. nurturant–involved (F)	−0.13 **	−0.11 **	−0.14 **	−0.22 **	−0.37 **	0.34 **	0.28 **	—	0.52 **
9	W3 Pa. nurturant–involved (A)	−0.13 **	−0.17 **	−0.21 **	−0.17 **	−0.20 **	0.26 **	0.47 **	0.48 **	—
Boys *M (SD)*		7.97 (6.02)	9.00 (7.08)	10.18 (7.06)	7.22 (5.99)	7.78 (6.10)	3.65 (0.80)	3.31 (0.97)	3.57 (0.80)	3.20 (0.92)
Girls *M* (*SD)*		8.11 (6.23)	9.75 (7.48)	10.16 (7.58)	6.70 (5.54)	7.29 (6.54)	3.74 (0.79)	3.42 (0.97)	3.76 (0.77)	3.38 (0.93)

Note. Pa. nurturant–involved = Paternal nurturant–involved parenting; (F) = father report; (A) = adolescent report; Lower diagonal = correlation matrix for the data of boys; Upper diagonal = correlation matrix for the data of girls; * *p* < 0.05, ** *p* < 0.01.
